# Community-wide patterns in pollen and ovule production, their ratio (P/O), and other floral traits along an elevation gradient in southwestern China

**DOI:** 10.1186/s12870-023-04433-2

**Published:** 2023-09-14

**Authors:** Shristhi Nepal, Judith Trunschke, Zong-Xin Ren, Kevin S. Burgess, Hong Wang

**Affiliations:** 1 Key Laboratory for Plant Diversity and Biogeography of East Asia, Kunming Institute of Botany, Chinese Academy of Sciences, Kunming, China; 2 University of Chinese Academy of Sciences, Beijing, China; 3 Nature Conservation and Landscape Ecology, Faculty of Environment and Natural Resources, University of Freiburg, Freiburg, Germany; 4 Department of Biology, College of Letters and Sciences, Columbus State University, University System of Georgia, Columbus, GA 31907-5645 USA

**Keywords:** Alpine, Floral traits, Ovule number, Phylogeny, Pollen number, Pollen-ovule ratio (P/O)

## Abstract

**Background:**

As the male and female gametophytes of flowering plants, pollen and ovules largely determine the upper and lower boundaries of plant reproductive success. It is commonly predicted that pollen and ovule number per flower should increase, and pollen-ovule ratio (P/O) per flower should decrease with increasing elevation in response to a more stochastic pollination environment. Here, we aimed to determine the response of pollen number, ovule number, and P/O to other floral traits and elevation gradients for 84 insect-pollinated herbaceous flowering plant species in five sub-alpine and alpine communities (2709 to 3896 m a.s.l.) on Yulong Snow Mountain, southwestern China.

**Results:**

Six floral traits, including P/O, floral display area, flower number, tube depth, flower shape, and pollen presentation, were highly correlated with pollen and ovule number per flower. With increasing elevation, pollen number and P/O per flower increased marginally and significantly, respectively; ovule number per individual, flower number per individual, stigma stamen separation, and inflorescence height decreased significantly. However, ovule number per flower and other floral traits (i.e., floral display area, tube depth, stigma height, stamen height, and pollen and P/O per individual) did not change with elevation. We detected significant phylogenetic signals for pollen number, ovule number, and P/O, suggesting that these traits may be highly conserved and with limited response to changing environmental conditions.

**Conclusions:**

Results revealed patterns of plant reproductive character evolution along elevation gradients and the potential factors governing their spatial variation in high-elevation environments. Plant species at high elevations are more likely adapted to cross-pollination, indicated by increased P/O per flower at high elevations on Yulong Mountain. Combined effects of phylogenetic history and plant-pollinator interactions should determine plant trait evolution.

**Supplementary Information:**

The online version contains supplementary material available at 10.1186/s12870-023-04433-2.

## Introduction

Pollen and ovule numbers can influence the dynamics of pollination and fertilization and set the upper and lower boundaries of seed production. Consequently, the amount of pollen and ovules per flower varies greatly among angiosperms [1–3]. For example, cleistogamous species can produce ~ 100 pollen grains per anther, while wind-pollinated plants may have more than 70,000 [4]. Similarly, the number of ovules per flower can range from one to several hundred [4, 5]. These traits are the key components of each flower’s male and female function and are thus selected to maximize a plant’s overall fitness [6, 7]. The resources allocated to pollen and ovule formation may vary greatly between species. Therefore, the numeric relationship between male and female gametes is expected to result from an evolutionary adjustment that balances the marginal fitness returns of increasing pollen and ovules through seed siring and production [8].

Pollen number per flower is positively related to the number of ovules per flower [9, 10], although the number of pollen grains is generally two to six orders of magnitude larger than the number of ovules per flower [11]. This discrepancy is due in part to an oversupply of pollen needed to account for inefficiencies in pollen transfer from one flower to another during the pollination process [12, 13]. For example, less than 1% of all pollen produced per flower in animal-pollinated plants reaches a conspecific stigma due to the loss during the pollen transfer [11, 14]. Generally, higher pollen production is expected when pollen transfer is more stochastic [3, 5]. Accordingly, outcrossing species produce more pollen than selfing [1, 15], and wind-pollinated species have more pollen than animal-pollinated ones [16, 17]. Positive directional selection for ovule number could also occur when pollination becomes more unpredictable [2, 5, 18]. The corresponding variation in the pollen-ovule ratio (P/O) is hypothesized to reflect variation in pollination efficiency. As such, P/O may indicate a plant’s breeding system [1, 19] and provide insight into the ecological and evolutionary mechanisms governing its variation among flowering plants.

The high variation in pollen number, ovule number, and P/O documented among flowering plants may be due to several biotic and abiotic factors acting on mate competition and pollen fate [5, 20]. Biotic factors such as breeding system, i.e., outcrossing versus selfing [1], pollination syndrome [17, 21], floral rewards [22], and flower biomass [3] can all have direct effects on pollen and ovule production and hence P/O. Alternatively, abiotic factors (i.e., water availability, temperature, and light) associated with elevational gradients can have a direct impact on traits such as plant size [22–24], leaf morphology [24], flower size [23], and flower longevity [25]. To compensate for the adverse reproductive fitness effects in highly stochastic pollination environments, plants at higher elevations may invest relatively more into reproductive structures compared to other traits in response to a low pollinator diversity, abundance, and activity [26, 27]. For example, plasticity for elongated floral longevity is higher for alpine plants than at lower elevations [25]. For many plant species, we expect variation in pollen number, ovule number, and P/O to be influenced by biotic factors acting differently along elevation gradients, although this source of variation is rarely tested.

Pollen number, ovule number, and P/O may be subject to stronger positive selection with increasing elevation [5, 28]. For example, as pollen transfer becomes more uncertain in upper elevations compared to the lower [17, 21], ovule oversupply should increase concomitantly with increasing elevation [3, 5, 29]. Flowers with a higher number of ovules may, in turn, have higher stigmatic pollen deposition, thus enabling them to produce more seeds than those with smaller ovule numbers [28]. In addition, because the number of ovules increases with flower size, selection for larger flowers may also be expected at higher elevations to further mitigate competition for scarce pollinator events [30, 31]. Although such floral traits are expected to vary for plant species at high elevations, the interactive effects of floral traits and elevation on pollen number, ovule number, and P/O are rarely empirically tested.

To fully understand the effects of the biotic and abiotic environment on the evolution of pollen and ovule number, as well as for P/O per flower/individual and other floral traits, their dispersion across the phylogeny (i.e., an estimation of their phylogenetic signal) should be determined [3]. This is important because plant morphological traits that share a phylogenetic history may constrain evolutionary responses to environmental selection [32, 33]. If the measured trait values are phylogenetically structured, they should produce co-occurrence patterns that are distinctively different among different plant communities [34]. Species within a community structured by environmental filtering, such as along elevation gradients, should have similar adaptations to a particular habitat, resulting in plant traits that are conserved between species [34]. On the other hand, for communities structured by strong competition between closely related species, phylogenetic overdispersion of traits can be expected, which may result in a divergence of species traits [35]. Testing the phylogenetic signal of reproductive traits can help to determine taxonomic relationships among closely related species [32] and identify potential constraints on the relationship between such traits and ecological factors.

Using comparative phylogenetic methods, we explored variation in the production of male and female gametes and their ratio at the flower, individual, and species level along an elevation gradient on Yulong Snow Mountain, southwestern China. In addition, variation in floral traits (i.e., floral display area, tube depth, stamen height, stigma height, stigma stamen separation, flower number, and inflorescence height) was evaluated across a range of insect-pollinated herbaceous flowering plant species. Specifically, we address the following questions: (1) How do pollen and ovule number and P/O vary with floral traits between different communities? (2) Do pollen and ovule number and P/O vary along an elevation gradient? (3) Is there a significant phylogenetic signal for pollen and ovule number, P/O, and floral traits? We predict that flowering plant species in higher-elevation communities will produce higher pollen and ovule numbers per flower to compensate for a more unpredictable pollination environment than in low-elevation communities. We also predict strong phylogenetic signals for some key reproductive traits of sub-alpine/alpine flowering plant species, suggesting their capacity for adaptive evolution.

## Materials and methods

### Study sites and flower community sampling

**Fig. 1:**
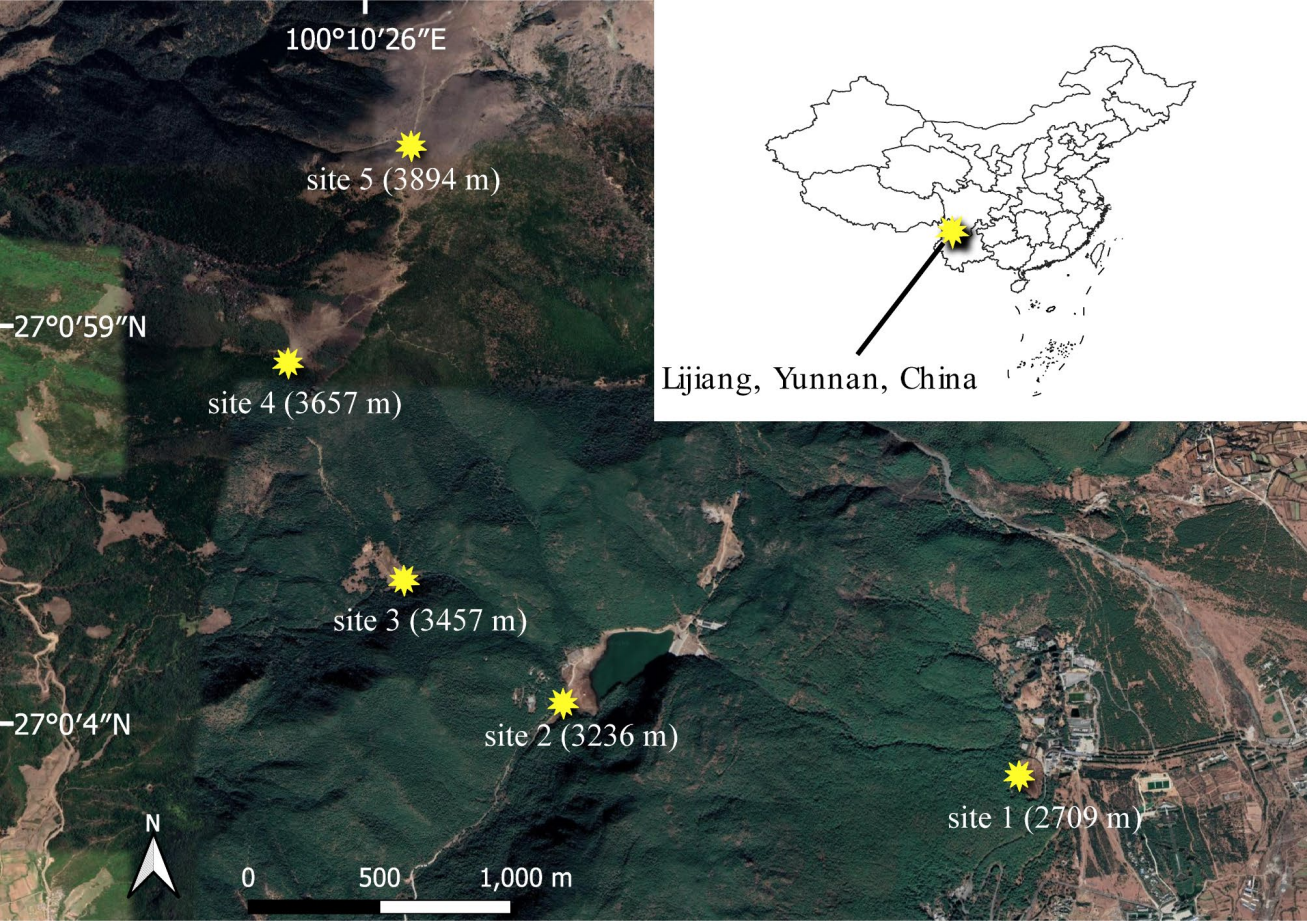
Map of the study area showing the five sub-alpine/alpine meadow communities along an elevation gradient from 2709 m a.s.l. (site 1) to 3894 m a.s.l. (site 5) on Yulong Mountain, southwestern China. Google Satellite Image exported from QGIS software

The study was conducted on Yulong Snow Mountain (27°00′ N, 100°10′ E) in the Himalayan-Hengduan Mountains region, southwestern China. This region is recognized as one of the world’s biodiversity hotspots [36], and Yulong Mountain hosts a high diversity of sub-alpine and alpine flowering plants with more than 2815 species [37]. The vegetation type is characterized by pine forests at low elevations, *Abies-Rhododendron* forests at mid-elevations, and dwarf *Rhododendron* forests at high elevations. The region is known for its warm and rainy monsoon season, from May to October, and a colder and drier period marked by occasional snow storms from November to April. The average temperature and relative humidity in the two growing seasons from June to September (2019 and 2020) were 13.5 °C and 85.2%, respectively, with an average precipitation of 11.2 mm in 2019; precipitation data for 2020 was lacking. We selected five different sub-alpine and alpine meadow communities ranging from 2709 to 3896 m a.s.l., spaced approximately 200–500 m a.s.l. apart on the eastern slope of the mountain (Fig. 1, Table S1). The three lowest sites are located within the Lijiang Forest Biodiversity National Observation and Research Station, Kunming Institute of Botany, Chinese Academy of Sciences (CAS). All except the lowest elevation community are extensively grazed by cows, horses, and yaks at lower elevations during the growing season, whereas yaks are the sole grazers at high elevations. All data (including flower buds for quantifying pollen and ovule production and flowers for various floral trait measurements) were collected from June to August over three consecutive flowering seasons (2019 to 2021).

We collected data for 84 species belonging to 57 genera and 23 families across the five different communities. Since some species occur at multiple elevations, we recorded data regarding each species, considering each community independently with a total of N = 24 (site 1), N = 40 (site 2), N = 22 (site 3), N = 22 (site 4), and N = 22 (site 5). In this way, we treated each population separately for species with populations in multiple communities (i.e., spanning elevation). Our sampling represents the majority of the flowering plant species within the study area. Given that we expected pollinator availability along the elevation gradient to impact plant reproductive traits, we focused our sampling regime on insect-pollinated herbaceous species. Dominant families include Asteraceae (14 spp.), Lamiaceae (9 spp.), Ranunculaceae (7 spp.), Campanulaceae (6 spp.), and Fabaceae (5 spp.).

All plant species studied and collected in the study area are not listed as protected or endangered. All fieldwork and collections were permitted by the Lijiang Forest Ecosystem National Observation and Research Station and the Special Foundation for National Science and Technology Basic Research Program of China (2021FY100200). They followed the guidelines and legislation of the Kunming Institute of Botany, Chinese Academy of Science, Yunnan Government, and the Government of China, as well as the rules of the Convention on the Trade in Endangered Species of Wild Fauna and Flora (https://www.cites.org/). Voucher specimens of all the species (from *Anemone rivularis*: SN10078 to *Viola biflora* var. *rockiana*: SN10207 from the Yulong Snow Mountain; Table S2) were identified by Shristhi Nepal and deposited in the herbarium of the Kunming Institute of Botany (KUN), Chinese Academy of Sciences, Kunming, China.

### Quantification of pollen and ovule production

To quantify pollen and ovule production per flower for each species, we collected at least ten mature flower buds, close to opening but anthers not dehisced, each from a different individual. All plant species sampled in this study have hermaphroditic flowers; therefore, we collected pollen samples and ovule data from the same flowers. All flower buds from each species were preserved in separate vials in 70% ethanol. We then randomly selected five of the collected flower buds per species and took one stamen per bud, each placed in a separate 2 mL centrifuge tube containing 1 mL distilled water. Each anther was gently ground using a glass rod to release the pollen into suspension. 20 µL of each suspension was placed on a Haemocytometer (Neubauer improved Haemocytometer, MARIENFELD, Tiefe-depth pro-founder 0.10 mm), and the number of pollen grains was counted under a light microscope at 10 × magnification [38]. The number of pollen grains in the 20 µL suspension was multiplied by the amount of suspension prepared (1 mL). Total pollen production per flower was calculated by multiplying the number of pollen grains in one anther by the number of anthers per flower. Finally, for each population, we calculated the mean ± SE for pollen counts across the five bud samples per population.

The ovule number per flower was counted from the same buds as anthers by dissecting the ovary and counting all ovules under a stereo-microscope [38]. We estimated the mean ± SE of ovule production by averaging across the five bud samples per population. In species of Asteraceae, we considered all the pollen and ovules present within one flower head as the number of pollen and ovules per flower following Arroyo et al. [3]. The ratio of pollen to ovule production was then calculated by dividing the number of pollen grains per flower by the number of ovules per flower. We also calculated the total pollen number, ovule number, and P/O per individual by multiplying each value by the average number of flowers per individual for each species (see below).

### Quantification of floral traits

We measured inflorescence height and counted flower production (number of flowers per individual) for 15–30 individuals per species directly in the field. For each individual, we measured 2–3 fresh, fully opened flowers for a series of flower morphological traits, including floral display area (the product of the vertical and horizontal distance between the two tips of the corolla), tube depth (length from the base of the tube to the tube opening), stamen height (length from the base of the stamen and the tip), stigma height (length from the base of the stigma to the tip), and stigma-stamen separation (distance between the tip of stigma and the anther tip). We measured floral traits using either (1) digital measurement of photographs of the flowers (front and side views) with the digital imaging software ImageJ 1.38e (http://rsbweb.nih.gov/ij/) [39] or (2) direct measurement of freshly collected flowers in the lab using a digital calliper with 0.01 mm precision. Using the same two methods, we also recorded a series of floral categorical (i.e., qualitative) traits such as flower shape (open/fused), flower cluster (solitary/clustered), flower symmetry (actinomorphic or zygomorphic), and pollen presentation (open/enclosed).

### Phylogeny construction

We constructed a phylogenetic tree using the function phylo.maker from the R package “V.Phylomaker” to determine the phylogenetic relationships among the studied plant species. We considered the GBOTB.extended mega phylogeny in Newick format as the backbone, default option “scenario 3” [40], where the tip for a new genus is bound to the upper 1/3 of the family branch (i.e., the branch between the family root node and the basal node). Polytomies in the final phylogenetic tree were resolved randomly using the function multi2di, available in the R package “ape” [41].

### Data analysis

Based on Shapiro-Wilk’s normality test, all data were Log_10_ transformed to meet the assumptions of normality for all statistical analyses. To visualize the phenotypic trait differences across the different communities, we applied a non-metric multidimensional scaling (NMDS) using the function metaMDS from the *Vegan* package [42] to each species. The analyses were based on a Bray-Curtis distance matrix and were run for a maximum of 100 iterations. We considered the reduced-dimension representations of our data to be acceptable if NMDS stress scores were ≤ 0.2 [43, 44]. We used the package ggplot2 to generate NMDS plots with confidence ellipses for each community [45].

We ran a correlation analysis (CA, Table S3) and principal component analysis (PCA) to investigate the correlation between phenotypic traits across the different communities. To further determine whether variation in pollen and ovule number and P/O significantly co-vary with different trait expression of morphological floral traits and elevations, we performed both a non-corrected and a phylogenetically-corrected analysis using Ordinary Least Square (OLS) regression and Phylogenetic Generalized Least Square (PGLS) regression, respectively. The main aim of comparing OLS and PGLS regression was to test the importance of phylogenetic relationships among the studied species and traits. PGLS were estimated using Pagel’s lambda (λ) transformation and a Brownian model (λ = 1) in the r package CAPER 3.1.3 [46]. In our model, we first used pollen number per flower as the response variable and all traits individually as the source of variation. Secondly, we used the ovule number per flower as the response variable and all traits individually as the source of variation. Third, we set all the quantitative traits separately as the response variable and elevation (i.e., community) as the source of variation. We used *p*-values and AIC values from the analysed models to compare the non-corrected and phylogenetically corrected models [3, 47, 48]. To test the effects of the categorical traits on pollen number and ovule number per flower, we performed Phylogenetic ANOVA and ANOVA, respectively.

We estimated the phylogenetic signal for all the traits to determine if the variation in trait expression among species correlates with their phylogenetic relationships. We used Pagel’s λ and Blomberg’s K to estimate phylogenetic signals, i.e., non-random trait evolution under a Brownian motion model [49, 50]. As Pagel’s λ and Blomberg’s K differ in their methods for testing phylogenetic signals, we use both estimates to ensure that our interpretation of the patterns found in our plant community is accurate [51]. The objective was to detect possible inconsistent results between co-flowering species across different communities. Pagel’s λ is typically used for community studies and Blomberg’s K for closely related species [49, 50, 52]. For Pagel’s λ, 0 indicates no phylogenetic signal, while 1 indicates a strong phylogenetic signal. Similarly, for Blomberg’s K, 0 indicates little or no phylogenetic signal, and 1 indicates a strong phylogenetic signal. Pagel’s λ and Blomberg’s K were calculated in the R package GEIGER [33]. Their significance was tested against a null distribution generated by 1000 random permutations of the tips of the phylogeny using the Picante package [53]. The overall data analysis was conducted in R version 4.0.2 [54].

## Results

### Pollen number, ovule number, and P/O per flower/individual, and floral traits

Pollen and ovule number, and P/O per flower, varied extensively among the 84 studied species. The average pollen number, ovule number, and P/O per flower were 432629.27 ± 112817.64, 65.97 ± 9.53, and 67412.01 ± 27536.19, respectively. Pollen production per flower ranged from 426.00 ± 31.33 (*Geranium nepalense*) to 7713000.00 ± 232384.70 (*Cynoglossum amabile*), and ovule production per flower ranged from one (*Polygonum macrophyllum*) to 830.4 ± 40.35 (*Inula helianthus*-*aquatilis*). P/O per flower ranged from 57.56 (*Geranium nepalense*) to 1928250.00 (*Cynoglossum amabile*). Furthermore, on an individual level, there was substantial variation observed among the studied species in terms of mean pollen number (81438170.8 ± 53355071.85), ovule number (856.96 ± 150.54), and P/O (19961993.81 ± 13343139.27). The studied species also varied significantly in mean floral display area (310.34 ± 35.74), stigma stamen separation (2.37 ± 0.37), flower number (40.36 ± 9.96), and inflorescence height (22.09 ± 1.25).

Our NMDS analysis converged on a two-dimensional solution with an acceptable level (non-metric fit R² = 0.96; linear fit R² = 0.83; stress = 0.19), and all the communities formed different clusters of points with a large amount of overlap on the NMDS plot (Figure S1). The correlation analysis (CA) and the principal component analysis (PCA) are shown in Table S3 and Figure S2, respectively. The first axis in the PCA (i.e., Dim1) accounts for 31% of the total variance, with contributions by stigma height, stamen height, stigma-stamen separation, and tube depth, followed by contributions of the floral display area and flower number per individual on community 1 (2709 m elevation site, see Figure S2). The second axis (i.e., Dim 2) accounts for 22.5% of the total variance, with contributions by ovule number on community 2 (3236 m elevation site) and following contributions by pollen number and P/O on community 1.

### Effects of phenotypic traits on pollen and ovule production

**Fig. 2:**
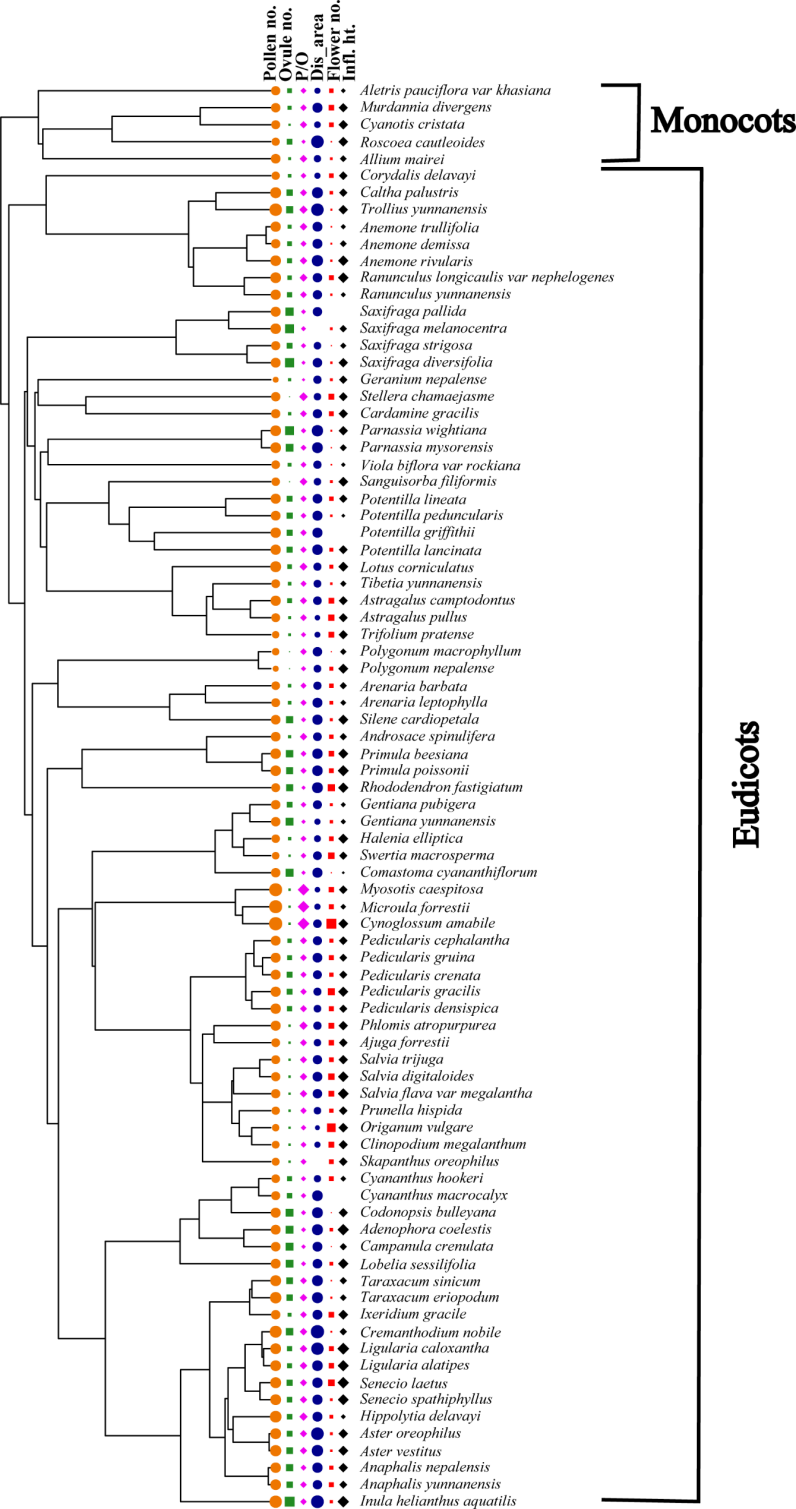
Phylogenetic relationships of the studied species and the dispersion of their quantitative traits. Shown are pollen number (Pollen no.), ovule number (Ovule no.), P/O per flower (P/O), floral display area (Dis_area), flower number (Flower no.), and inflorescence height (Infl. ht.). Relative values for each respective trait are reflected by the size of the symbol; the larger the symbol the higher the value

**Fig. 3:**
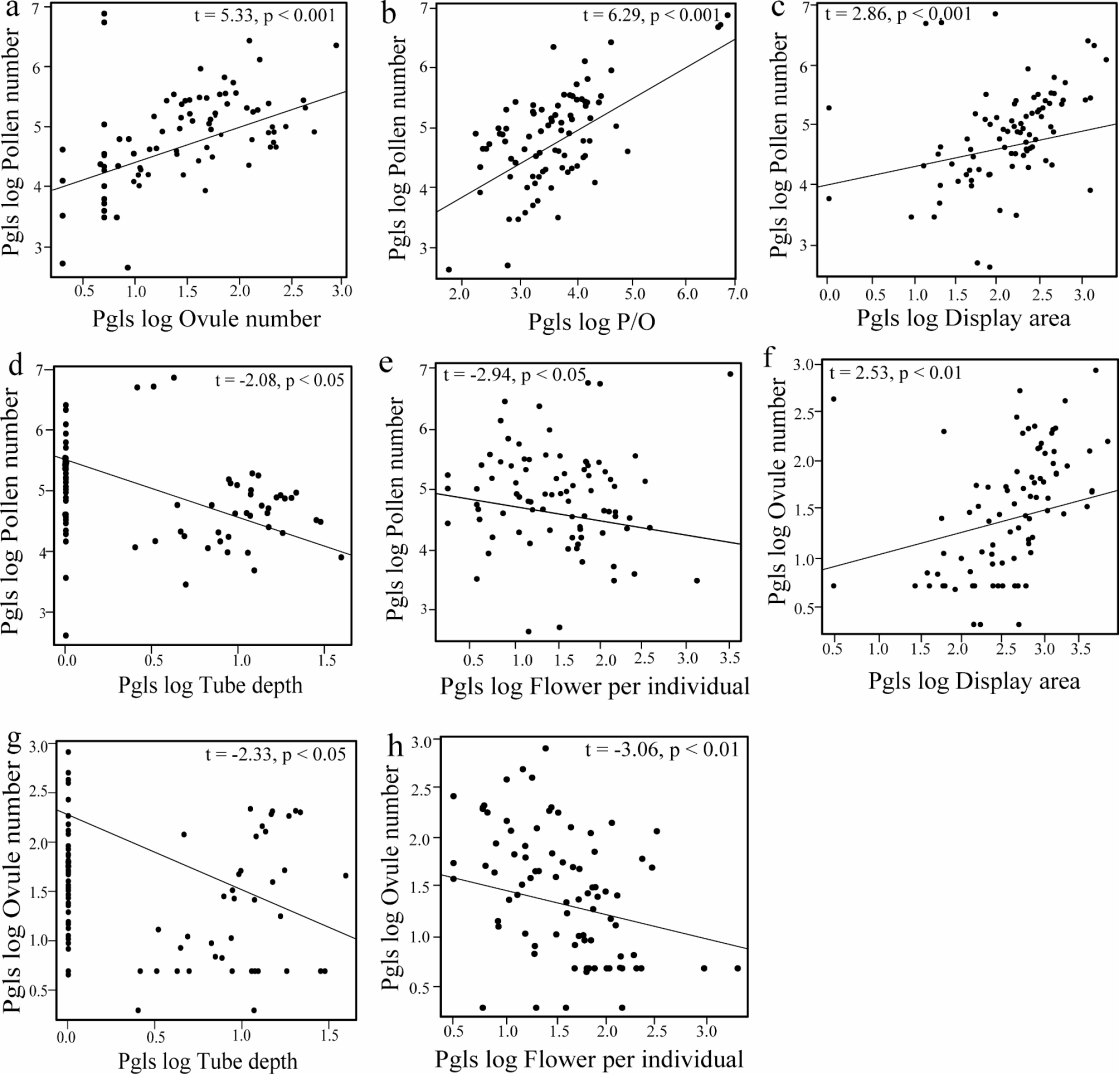
The relationship between pollen and ovule number per flower (**a**) and their relationship with P/O (**b**), floral display area (**c** and **f**), flower tube depth (**d** and **g**), and flower number per individual (**e** and **h**), respectively. Lines were fitted according to the best-fit PGLS model; values of all traits were log-transformed

**Fig. 4:**
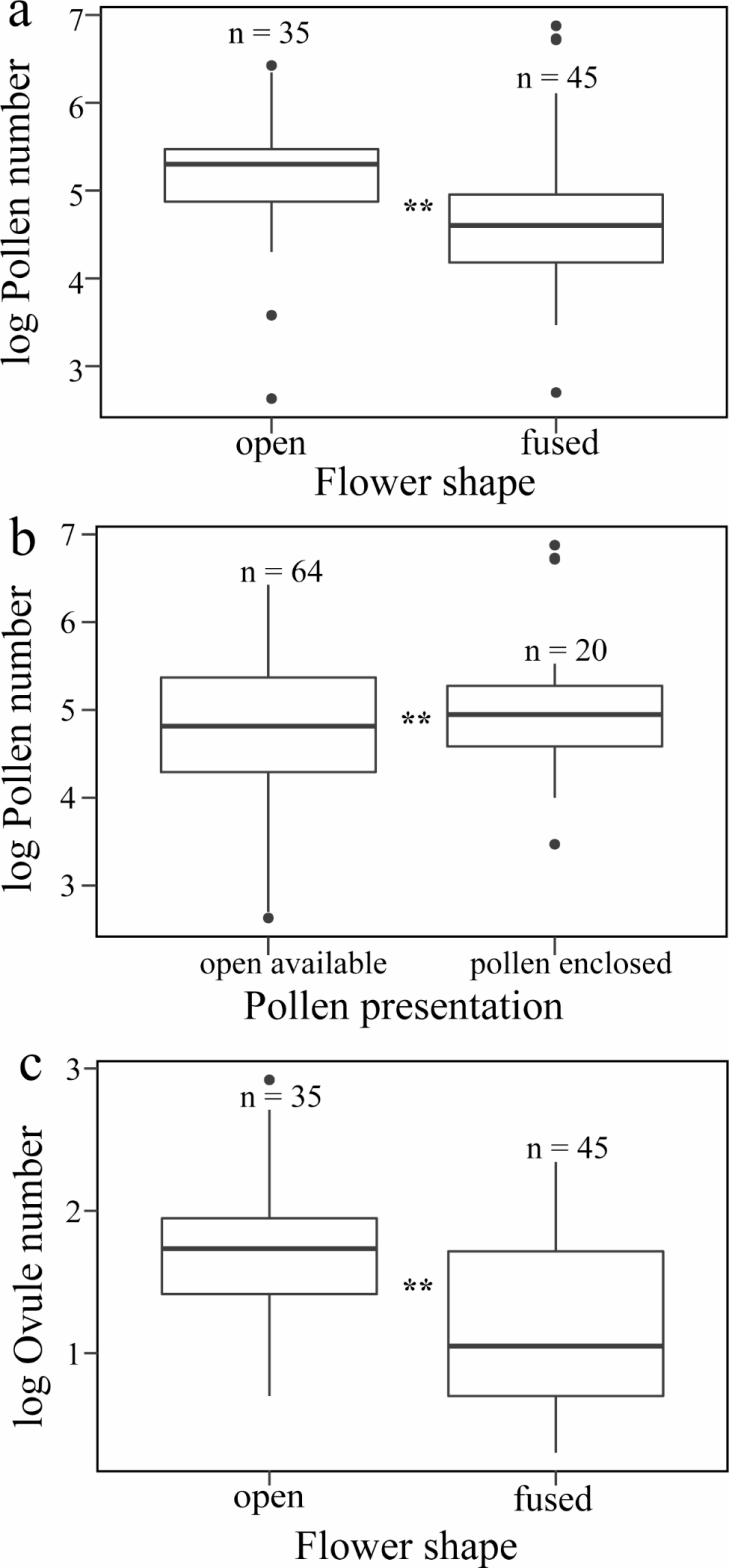
Pollen and ovule number per flower and their relationship with qualitative floral traits. (**a**) x-axis: flower shape; y-axis: pollen number per flower, (**b**) x-axis: pollen presentation; y-axis: pollen number per flower, and (**c**) x-axis: flower shape; y-axis: ovule number per flower. All response variables were log-transformed

Figure 2 shows the constrained phylogenetic tree for all the species with pollen number, ovule number, P/O, and floral traits. Our phylogenetic tree reflected the unequal distribution of all the measured traits throughout the tree, and it was the basis for comparing the analysis with and without considering the phylogeny. The best-performing models with pollen number as a response variable and the floral traits as predictor variables in both the PGLS and OLS regression model showed relatively consistent results with similar significant *p*-values (Table S4). However, based on the Akaike Information Criterion (AIC), the best-fit model was PGLS, which had consistently lower AIC values than those for OLS except when stamen height was used as a predictor (Table S4). Both models suggest a strong positive relationship between pollen number per flower with ovule number per flower, P/O per flower, and floral display area (Table S4; Fig. 3a-c). Pollen number per flower showed a significant negative relation with tube depth and with flower number per individual, the latter being only significant using PGLS (Table S4; Fig. 3d-e). The remaining phenotypic traits showed no association with pollen production per flower (Table S4). Both the phylogenetically corrected and the non-corrected models indicated that pollen number per flower was significantly related to categorical floral traits like flower shape and pollen presentation (Table S4; Fig. 4a-b), whereas flower cluster and flower symmetry did not significantly affect pollen number per flower (Table S4).

The best-performing models with ovule number as a response variable and floral phenotypic traits as predictor variables for both the PGLS and OLS regression showed relatively consistent significant results, although based on the lowest AIC value criteria, the PGLS models performed consistently better again except when stamen height was used as a predictor (Table S5). Both PGLS and OLS models showed that ovule number per flower had a significant negative relationship with P/O, flower number per individual, and tube depth. A significant positive relationship was found with floral display area (Table S5; Fig. 3f-h). The remaining traits did not significantly affect the ovule number per flower (Table S5). The phylogenetically corrected and non-corrected models showed that ovule number per flower was significantly related to flower shape (Fig. 4c) but not to flower cluster, flower symmetry, and pollen presentation (Table S5).

### Effect of elevation on the focal traits with and without phylogeny

**Fig. 5:**
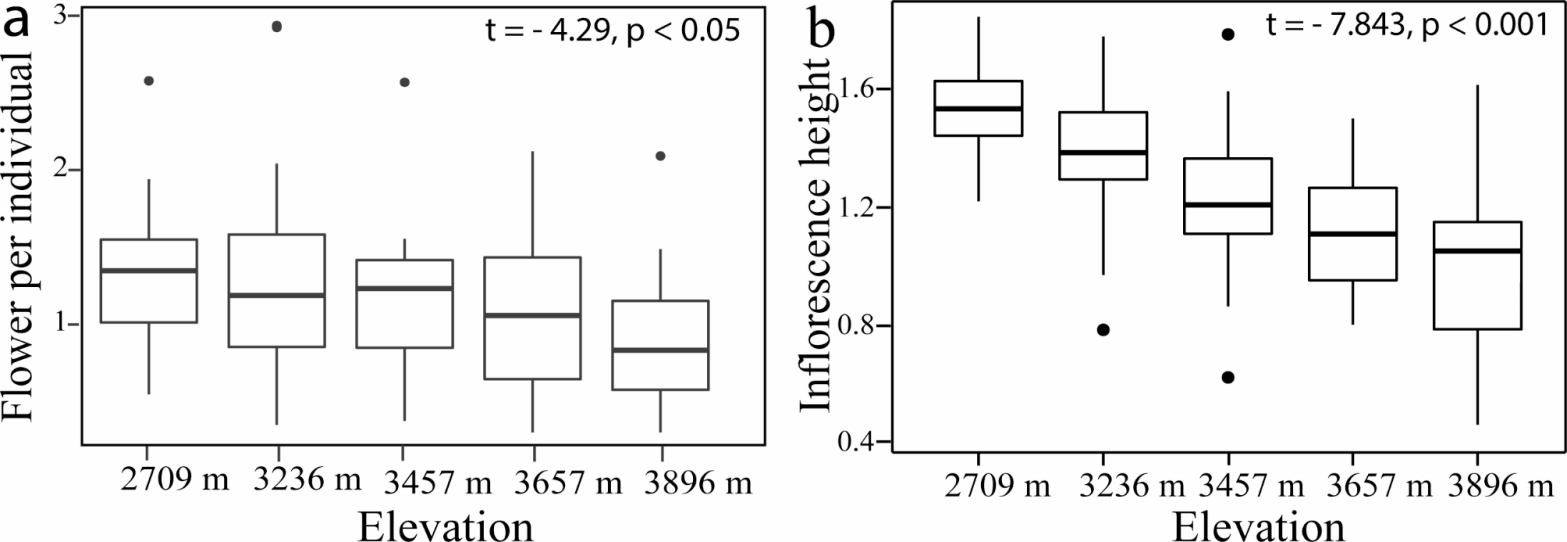
Non-phylogenetic analysis of five meadow communities located along an elevation gradient on Yulong Mountain, southwestern China. Plots representing a significant effect of elevation on the number of flowers per individual (**a**), and inflorescence height (**b**)

The best-performing models analysing the effect of elevation on pollen and ovule number, P/O, and phenotypic traits showed inconsistent results between the PGLS and OLS analyses, although phylogenetically corrected analysis in some traits improved the fit to a Brownian motion (λ = 1) model of evolution. Elevation patterns on P/O per flower, ovule number per individual, and stigma-stamen separation differed between the PGLS and OLS regression models (Table 1). When phylogeny was considered, P/O increased significantly with increasing elevation, whereas stigma-stamen separation decreased significantly (Table 1; Figure S3a-b); for both of these traits, the effect disappeared in the OLS model, which had higher AIC values (Table 1; Figure S3a-b). Further, ovule number per individual, flower number per individual, and inflorescence height significantly decreased with increasing elevation (lower AIC values in the OLS model; Table 1). However, only the latter two response variables significantly decreased as elevation increased in both models (Table 1; Fig. 5a-b & Figure S3c). Elevation did not explain variation in any of the remaining traits regardless of using PGLS or OLS regression models (Table 1, Figure S3d-g).

**Table 1:** Maximum likelihood tests within a 95% confidence interval comparing phylogenetic generalized least square regression (**PGLS**) and ordinary least square (**OLS**) regression for a series of phenotypic traits on elevation. Significant effects at *p* < 0.05 are presented in bold

Quantitative traits	PGLS (Maximum likelihood) regression						OLS regression		
	AIC	BIC	logLik	t-value	*p*-value	AIC		t-value	*p*-value
Pollen number ~ Elevation	157.39	164.69	-75.70	1.90	**0.06**	207.18		0.02	0.98
Ovule number ~ Elevation	136.06	143.36	-65.03	-0.43	0.67	168.08		0.82	0.41
P/O ~ Elevation	146.02	153.32	-70.01	2.43	**0.01**	202.11		-0.72	0.48
Display area ~ Elevation	700.23	707.53	-347.12	0.85	0.40	134.14		-0.08	0.93
Tube depth ~ Elevation	52.38	59.67	-23.19	0.18	0.85	130.87		0.79	0.43
Stamen height ~ Elevation	205.95	213.25	-99.98	0.68	0.49	3.67		-0.63	0.53
Stigma height ~ Elevation	209.38	216.67	-101.69	-0.11	0.91	32.95		-0.72	0.47
Stigma stamen separation ~ Elevation	83.04	90.33	-38.52	-2.79	**< 0.05**	861.96		0.74	0.45
Flower number ~ Elevation	179.65	186.95	-86.83	-2.72	**< 0.05**	128.96		-2.41	**< 0.05**
Inflorescence height ~ Elevation	81.68	88.97	-37.84	-7.50	**< 0.001**	-3.73		-7.84	**< 0.001**
Pollen per individual ~ Elevation	322.28	329.57	-158.14	-0.74	0.45	232.67		-1.36	0.17
Ovules per individual ~ Elevation	241.33	248.62	-117.66	-2.35	**< 0.05**	172.52		-1.25	0.21
P/O per individual ~ Elevation	291.15	298.44	-142.57	-0.75	0.45	253.27		-1.75	0.08

### Phylogenetic signal

The phylogenetic signal for the quantitative traits was more robust for Pagel’s lambda (λ) compared to Blomberg’s k (Table 2). Focal traits such as pollen number, ovule number, P/O, and pollen presentation showed a high and significant phylogenetic signal (λ ranged from 0.93 to 0.96), whereas the signal was moderate to weak (0.89–0.31) for traits such as floral display area, stamen height, stigma height, stigma stamen separation, flower number, and flower symmetry (Table 2). Flower shape had the weakest phylogenetic signal (λ = 0.09), whereas the phylogenetic signals for tube depth and flower cluster were 1.01 and 1.00, respectively, consistent with Brownian motion evolution. Inflorescence height was the only trait that did not show a significant phylogenetic signal (See details in Table 2).

**Table 2:** Phylogenetic signals of quantitative and qualitative traits estimated by Pagel’s lambda and Blomberg’s k. Significant effects at *p* < 0.05 are presented in bold

Quantitative traits (Log-transformed)	Pagel’s lambda		Blomberg’s k	
	λ	*p*-value	k	*p*-value
Pollen number	0.95	**< 0.001**	0.61	**< 0.001**
Ovule number	0.93	**< 0.001**	0.48	**< 0.001**
P/O	0.95	**< 0.001**	0.60	**< 0.001**
Display area	0.62	**< 0.001**	0.31	**< 0.001**
Tube depth	1.01	**< 0.001**	1.05	**< 0.001**
Stamen height	0.74	**< 0.001**	0.39	**< 0.001**
Stigma height	0.88	**< 0.001**	0.61	**< 0.001**
Stigma stamen separation	0.31	**0.01**	0.26	**0.03**
Flower number	0.38	**< 0.001**	0.22	**< 0.05**
Inflorescence height	0.05	0.67	0.17	0.05
**Qualitative traits**	**log-likelihood**	**AIC**	**λ**	***p*** **-value**
Flower shape	-33.42	70.84	0.09	**< 0.05**
Flower cluster	-22.10	48.20	1.00	**< 0.05**
Flower symmetry	-29.14	62.27	0.89	**< 0.05**
Pollen presentation	-31.83	67.66	0.96	**< 0.05**

## Discussion

### Floral trait variation

Using a multispecies approach, we investigated pollen and ovule production and their relation with a series of floral traits in five sub-alpine and alpine communities across an elevation gradient. We found that pollen and ovule production per flower was higher in open flowers (i.e., flowers without tubes where pollen presentation is more open) than in fused flowers. This result is not surprising since plants with generalized flowers (e.g., Asteraceae, Rosaceae) typically attract a diverse pollinator guild [55]. Here, higher pollen and ovule production ensure reproductive success under low pollinator fidelity, accompanied by higher pollen dislodging, compared to more specialized flowers (e.g., Lamiaceae), which are more frequently pollinated by specialized pollinators [55–57].

In addition to floral shape and presentation, we found a significant positive correlation between flower size and pollen and ovule number per flower. In general, in our communities, larger flowers tended to have higher male and female gamete production than smaller flowers. These results are consistent with a widely reported phenomenon; significant positive correlations between flower size and pollen number, and ovule number per flower [30, 58, 59]. In contrast, we found a significant negative relationship between flower production per individual and pollen number, and ovule number per flower. Collectively, these results suggest that to maximize fertilization success, plants in our sub-alpine and alpine communities may be allocating resources to a few but large flowers with high gamete production [60], although this relationship remains to be experimentally tested.

### Floral traits vary with elevation

We found that pollen production and P/O per flower increased with increasing elevation only after accounting for phylogeny but did not affect the production of ovules per flower with or without phylogeny included in the analysis. This result is similar to previous studies, e.g., Cunha et al. [48], where increased pollen production across increasing latitude is more significant with phylogeny, which may indicate an evolutionary response to an unpredictable stochastic pollination environment. Given that pollen number, and P/O per flower, varied with elevation before and after taking phylogeny into account, variation in pollen production may be more indicative of a response to the environment rather than an evolutionary adaptation. Further, our findings that increased pollen production, and hence P/O, but no concomitant changes in ovule number with the increasing elevation may indicate that pollinator dependence and pollination efficiency have a greater effect on the evolution of pollen production compared to ovule production [61]. The pollinator guild of the Himalayan-Hengduan Mountains region is dominated by hymenopteran visitors at higher elevations [55, 62], whereas in other regions, bee abundance and richness decrease with increasing elevation and are replaced by flies as the dominant visitors [63]. Many of the herbaceous flowering plant species in our study sites are insect-pollinated [55, 62], and as such, pollinator dependence and pollination efficiency may play a large role in the trends for pollen production and P/O found in our study, although this source of variation remains to be empirically tested in the field.

At the individual level, pollen production and P/O did not significantly change with increasing elevation, although ovule production significantly decreased when phylogeny was considered. This result contrasts with our findings for pollen production and P/O per flower, suggesting that plants, on average, produce fewer flowers with higher pollen numbers in more stressful habitats to balance the adverse effects of environmental factors on fertilization with increasing elevation. Similarly, the fact that we found a decrease in ovule production per individual, but not per flower, with increasing elevation, may also be the direct effect of plants having fewer flowers per individual at higher elevations. Given that overall ovule production at the flower level did not change across the elevation gradient in our study, future studies should test if the average number of ovules per flower might be an adaptive response to ensure successful fertilization in more unpredictable pollination environments at higher elevations.

Elevational variation in mating systems [64], pollination modes [65, 66], and specialization [67] all suggest that plants can adapt to unpredictable pollination environments. A high degree of unpredictability among species interactions, particularly at higher elevations, could favour traits that reduce reliance on mutualisms or increase pollinator predictability in harsher weather conditions [68, 69]. Considering potential reductions in pollinator diversity, abundance, and activity, we predicted that pollen and ovule production per flower would be greater in our higher elevational communities. Our results on animal-pollinated flowering plants support this prediction, indicating that species at high elevations on Yulong Mountain may compensate for decreased plant-pollinator interactions [70] by producing more pollen grains per flower.

Our study was limited to the sub-alpine and alpine communities across one elevation gradient (2709 m a.s.l. to 3896 m a.s.l.) without covering different environments across multiple mountain ranges. As such, it may explain why we did not find a strong significant relation between pollen production and elevation and a lack of support for concomitant increases in ovule production. This last finding, in particular, is in stark contrast to the ovule bet-hedging hypothesis by Arroyo et al. [3], which, according to Burd et al. [2], predicts the unpredictability of the pollinator environment may select for an increase in ovule number. They hypothesized that plants that package more ovules per flower might take advantage of rare pollination events through higher stigmatic pollen deposition, enabling them to produce more seeds than plants with fewer ovules per flower [28, 48]. Future studies should include a larger environmental gradient to more fully address this hypothesis by comparing plant traits within communities from lower elevation regions (sub-tropical/temperate) to those at high (sub-) alpine sites.

Finally, we found the number of flowers per individual had a strong negative relationship with elevation, as did the distance between the stigma and stamen tips. These findings support previous evidence that plants produce fewer flowers at high elevations (e.g. alpine regions) and shorten the distance between the stigma and stamen tips to ensure successful fertilization through self-pollination [3, 48]. Two findings from our study, an increased pollen number and P/O per flower in higher alpine species indicating a potentially higher outcrossing rate and a closer distance between stigma and stamen tips, may increase the potential for selfing. However, since we have not conducted bagging/hand-pollination to test the breeding systems for all the plants in this study, we cannot draw conclusive results to show the trends of breeding system change along the elevation. Here, at high-elevation sites like those found in our study, plants might reduce investment in flower production by producing structural components such as head stalks, involucres, and receptacles. In addition, they may have fewer flowers with higher pollen production [24], longer flower longevity [25] and increased stigma receptivity [71]. Although not empirically tested in this study, these strategies can directly contribute to successful fertilization and seed production and should be investigated further. We should also consider that in this region, cold-adapted social bumblebees are dominant pollinator groups in high elevations [62], and bumblebees play a key role in shaping plant reproductive strategy. Indeed, high-elevation populations of bumblebee-pollinated *Incarvillea mairei* also have high cross-pollination rates compared with lower-elevation populations [72].

### Phylogenetic signal on floral trait variation

In addition to determining the contribution of elevation to explaining floral trait variation in our plant communities, we found evidence of a strong phylogenetic signal in pollen number, ovule number, and P/O in the studied species. Further, floral traits such as flower cluster, flower symmetry, and pollen presentation showed strong significant phylogenetic signals, although it was only moderate to absent for floral traits. These results suggest that, in most cases, the adaptive capacity of the floral traits for the species investigated may be constrained by their evolutionary history regardless of their ecological selection regime (i.e., elevation or pollinator community). Phylogenetic signal has shown to vary among 16 quantitative traits in Bignoniaceae [73], be strong for flower size in Cistaceae [74], weak to absent for flower colour across multiple species [75], and moderate for ovule number and flower biomass [3]. Little is known, however, about how evolutionary constraints on floral traits, such as pollen and ovule number, vary among species and communities that span elevation. Although our sampling regime did not allow us to test this source of variation directly, future studies should investigate the phylogenetic dispersion of floral traits to see how such traits may directly or indirectly impact successful fertilization and seed production, which may, in turn, affect species establishment, persistence and community composition.

Our research on sub-alpine and alpine meadow species suggests that high-elevation insect-pollinated communities produce more pollen per flower, resulting in higher P/O yet overall reductions in inflorescence height, flower production, tube height, and the distance between stigma tip to stamen tip. Furthermore, flower traits such as the floral display size and tube depth are highly correlated with pollen and ovule production but not with elevation, suggesting a plausible mechanism driving the pollination efficiency hypothesis. Ours is the first study to investigate pollen and ovule production, and P/O, as a function of elevation and floral traits for the majority of herbaceous species on Hengduan Mountains region, southwestern China. Until the present study, pollen and ovule production among different species spanning communities has received little attention. Until now, most pollen and ovule production studies are limited to species-level [69, 76, 77] or family-level [58, 59, 78] comparisons. However, it is important to note that our study did not assess plant fitness, which we expect to be relatively high in the sub-alpine compared to alpine plant communities via increased visitation and pollen export [14, 78, 79]. Future experimental studies in other mountain communities should include bagged and hand-pollination experiments to test additional factors governing patterns of plant reproductive character evolution along elevational gradients.

### Electronic supplementary material

Below is the link to the electronic supplementary material.

Supplementary Material 1

## Data Availability

Additional information regarding tables and figures is included in Additional_file1.doc as a supplementary file.
