# Supplementary material for: Community-wide patterns in pollen and ovule production, their ratio (P/O), and other floral traits along an elevation gradient in southwestern China
Source: BMC Plant Biol. 2023 Sep 14;23:425. doi: 10.1186/s12870-023-04433-2 (PMC10500814; doi:10.1186/s12870-023-04433-2)
Supplement: Supplementary file 1 — Supplementary Material 1 [file 12870_2023_4433_MOESM1_ESM.docx]

**SUPPLEMENTARY FILES:**

**Table S1**: Details of the study sites, latitude, longitude, elevation, and the number of plant species studied.

| Transects | Latitude (N) | Longitude (E) | Elevation (m a.s.l.) | Species number |
| --- | --- | --- | --- | --- |
| Site-1 | 27°00′10″ | 100°12′05″ | 2709 | 24 |
| Site-2 | 27°00′09″ | 100°10′57″ | 3236 | 40 |
| Site-3 | 27°00′26″ | 100°10′25″ | 3457 | 22 |
| Site-4 | 27°00′59″ | 100°10′21″ | 3657 | 22 |
| Site-5 | 27°01′21″ | 100°10′21″ | 3896 | 22 |

**Table S2**: Species details representing collection ID, species name, their family, elevation (m a.s.l.) and study sites.

| **Collection ID** | **Species** | **Family** | **Elevation** | **Sites** |
| --- | --- | --- | --- | --- |
| SN10078 | *Anemone rivularis* Buchanan-Hamilton ex de Candolle | Ranunculaceae | 2709 | Site_1 |
| SN10079 | *Aster vestitus* Franchet | Asteraceae | 2709 | Site_1 |
| SN10080 | *Clinopodium megalanthum* (Diels) C. Y. Wu & Hsuan ex H. W. Li | Lamiaceae | 2709 | Site_1 |
| SN10081 | *Cyanotis cristata* (Linnaeus) D. Don | Commelinaceae | 2709 | Site_1 |
| SN10082 | *Halenia elliptica* D. Don | Gentianaceae | 2709 | Site_1 |
| SN10083 | *Inula helianthus-aquatilis* C. Y. Wu ex Y. Ling | Asteraceae | 2709 | Site_1 |
| SN10084 | *Ixeridium gracile* (Candolle) Pak & Kawano | Asteraceae | 2709 | Site_1 |
| SN10085 | *Ligularia caloxantha* (Diels) Handel-Mazzetti | Asteraceae | 2709 | Site_1 |
| SN10086 | *Lobelia sessilifolia* Lambert | Campanulaceae | 2709 | Site_1 |
| SN10087 | *Lotus corniculatus* Linnaeus | Fabaceae | 2709 | Site_1 |
| SN10088 | *Murdannia divergens* (C. B. Clarke) Brückner | Commelinaceae | 2709 | Site_1 |
| SN10089 | *Origanum vulgare* Linnaeus | Lamiaceae | 2709 | Site_1 |
| SN10090 | *Parnassia wightiana* Wallich ex Wight & Arnott | Celastraceae | 2709 | Site_1 |
| SN10091 | *Pedicularis crenata* Maximowicz | Orobanchaceae | 2709 | Site_1 |
| SN10092 | *Pedicularis gruina* Franchet ex Maximowicz | Orobanchaceae | 2709 | Site_1 |
| SN10093 | *Polygonum nepalense* Meisner | Polygonaceae | 2709 | Site_1 |
| SN10094 | *Potentilla griffithii* J. D. Hooker | Rosaceae | 2709 | Site_1 |
| SN10095 | *Primula poissonii* Franchet | Primulaceae | 2709 | Site_1 |
| SN10096 | *Prunella hispida* Bentham. | Lamiaceae | 2709 | Site_1 |
| SN10097 | *Ranunculus longicaulis var. nephelogenes* Edgeworth | Ranunculaceae | 2709 | Site_1 |
| SN10098 | *Sanguisorba filiformis* (J. D. Hooker) Handel-Mazzetti | Rosaceae | 2709 | Site_1 |
| SN10099 | *Senecio laetus* Candolle | Asteraceae | 2709 | Site_1 |
| SN10100 | *Senecio spathiphyllus* Franchet | Asteraceae | 2709 | Site_1 |
| SN10101 | *Trifolium pratense* Linn. | Fabaceae | 2709 | Site_1 |
| SN10102 | *Adenophora coelestis* Diels | Campanulaceae | 3236 | Site_2 |
| SN10103 | *Ajuga forrestii* Diels | Lamiaceae | 3236 | Site_2 |
| SN10104 | *Anaphalis nepalensis* (Sprengel) Handel-Mazzett | Asteraceae | 3236 | Site_2 |
| SN10105 | *Anemone demissa* J. D. Hooker & Thomson | Ranunculaceae | 3236 | Site_2 |
| SN10106 | *Anemone rivularis* Buchanan-Hamilton ex de Candolle | Ranunculaceae | 3236 | Site_2 |
| SN10107 | *Arenaria barbata* Franchet | Caryophyllaceae | 3236 | Site_2 |
| SN10108 | *Aster oreophilus* Franchet | Asteraceae | 3236 | Site_2 |
| SN10109 | *Astragalus camptodontus* | Fabaceae | 3236 | Site_2 |
| SN10110 | *Astragalus pullus* N. D. Simpson | Fabaceae | 3236 | Site_2 |
| SN10111 | *Caltha palustris* Linnaeus | Ranunculaceae | 3236 | Site_2 |
| SN10112 | *Cardamine gracilis* (O. E. Schulz) T. Y. Cheo & R. C. Fang | Brassicaceae | 3236 | Site_2 |
| SN10113 | *Cynoglossum amabile* Stapf & J. R. Drummond | Boraginaceae | 3236 | Site_2 |
| SN10114 | *Gentiana pubigera* C. Marquand | Gentianaceae | 3236 | Site_2 |
| SN10115 | *Geranium nepalense* Sweet | Geraniaceae | 3236 | Site_2 |
| SN10116 | *Halenia elliptica* D. Don | Gentianaceae | 3236 | Site_2 |
| SN10117 | *Ligularia alatipes* Handel-Mazzetti | Asteraceae | 3236 | Site_2 |
| SN10118 | *Lotus corniculatus* Linnaeus | Fabaceae | 3236 | Site_2 |
| SN10119 | *Myosotis caespitosa* C. F. Schultz | Boraginaceae | 3236 | Site_2 |
| SN10120 | *Origanum vulgare* Linnaeus | Lamiaceae | 3236 | Site_2 |
| SN10121 | *Parnassia mysorensis* F. Heyne ex Wight & Arnott | Celastraceae | 3236 | Site_2 |
| SN10122 | *Pedicularis cephalantha* Franchet ex Maximowicz | Orobanchaceae | 3236 | Site_2 |
| SN10123 | *Pedicularis densispica* Franchet ex Maximowicz | Orobanchaceae | 3236 | Site_2 |
| SN10124 | *Pedicularis gracilis* Wallich ex Bentham | Orobanchaceae | 3236 | Site_2 |
| SN10125 | *Pedicularis gruina* Franchet ex Maximowicz | Orobanchaceae | 3236 | Site_2 |
| SN10126 | *Phlomis atropurpurea* Dunn | Lamiaceae | 3236 | Site_2 |
| SN10127 | *Polygonum nepalense* Meisner | Polygonaceae | 3236 | Site_2 |
| SN10128 | *Potentilla lancinata* Cardot | Rosaceae | 3236 | Site_2 |
| SN10129 | *Potentilla lineata* Treviranus | Rosaceae | 3236 | Site_2 |
| SN10130 | *Primula beesiana* Forrest | Primulaceae | 3236 | Site_2 |
| SN10131 | *Primula poissonii* Franchet | Primulaceae | 3236 | Site_2 |
| SN10132 | *Prunella hispida* Bentham | Lamiaceae | 3236 | Site_2 |
| SN10133 | *Roscoea cautleoides* Gagnepain | Zingiberaceae | 3236 | Site_2 |
| SN10134 | *Salvia digitaloides* Diels | Lamiaceae | 3236 | Site_2 |
| SN10135 | *Salvia flava* var. *megalantha* Diels | Lamiaceae | 3236 | Site_2 |
| SN10136 | *Saxifraga diversifolia* Wallich ex Seringe in de Candolle | Saxifragaceae | 3236 | Site_2 |
| SN10137 | *Saxifraga pallida* Wallich ex Seringe in de Candolle | Saxifragaceae | 3236 | Site_2 |
| SN10138 | *Silene cardiopetala* Franchet | Caryophyllaceae | 3236 | Site_2 |
| SN10139 | *Skapanthus oreophilus* (Diels) C. Y. Wu & H. W. Li | Lamiaceae | 3236 | Site_2 |
| SN10140 | *Taraxacum sinicum* (D. Don) Candolle | Asteraceae | 3236 | Site_2 |
| SN10141 | *Tibetia yunnanensis* (Franchet) H. P. Tsui | Fabaceae | 3236 | Site_2 |
| SN10142 | *Trollius yunnanensis* (Franchet) Ulbrich | Ranunculaceae | 3236 | Site_2 |
| SN10143 | *Anaphalis nepalensis* (Sprengel) Handel-Mazzett | Asteraceae | 3457 | Site_3 |
| SN10144 | *Anemone rivularis* Buchanan-Hamilton ex de Candolle | Ranunculaceae | 3457 | Site_3 |
| SN10145 | *Astragalus camptodontus* | Fabaceae | 3457 | Site_3 |
| SN10146 | *Astragalus pullus* N. D. Simpson | Fabaceae | 3457 | Site_3 |
| SN10147 | *Caltha palustris* Linnaeus | Ranunculaceae | 3457 | Site_3 |
| SN10148 | *Cynoglossum amabile* Stapf & J. R. Drummond | Boraginaceae | 3457 | Site_3 |
| SN10149 | *Gentiana pubigera* C. Marquand | Gentianaceae | 3457 | Site_3 |
| SN10150 | *Halenia elliptica* D. Don | Gentianaceae | 3457 | Site_3 |
| SN10151 | *Ligularia alatipes* Handel-Mazzetti | Asteraceae | 3457 | Site_3 |
| SN10152 | *Lotus corniculatus* Linnaeus | Fabaceae | 3457 | Site_3 |
| SN10153 | *Pedicularis cephalantha* Franchet ex Maximowicz | Orobanchaceae | 3457 | Site_3 |
| SN10154 | *Pedicularis densispica* Franchet ex Maximowicz | Orobanchaceae | 3457 | Site_3 |
| SN10155 | *Phlomis atropurpurea* Dunn | Lamiaceae | 3457 | Site_3 |
| SN10156 | *Potentilla lancinata* Cardot | Rosaceae | 3457 | Site_3 |
| SN10157 | *Potentilla lineata* Treviranus | Rosaceae | 3457 | Site_3 |
| SN10158 | *Primula poissonii* Franchet | Primulaceae | 3457 | Site_3 |
| SN10159 | *Prunella hispida* Bentham | Lamiaceae | 3457 | Site_3 |
| SN10160 | *Sanguisorba filiformis* (J. D. Hooker) Handel-Mazzetti | Rosaceae | 3457 | Site_3 |
| SN10161 | *Taraxacum sinicum* Kitagawa | Asteraceae | 3457 | Site_3 |
| SN10162 | *Tibetia yunnanensis* (Franchet) H. P. Tsui | Fabaceae | 3457 | Site_3 |
| SN10163 | *Trollius yunnanensis* (Franchet) Ulbrich | Ranunculaceae | 3457 | Site_3 |
| SN10164 | *Anaphalis nepalensis* (Sprengel) Handel-Mazzett | Asteraceae | 3657 | Site_4 |
| SN10165 | *Anaphalis yunnanensis* (Franchet) Diels | Asteraceae | 3657 | Site_4 |
| SN10166 | *Androsace spinulifera* (Franchet) R. Knuth in Engler | Primulaceae | 3657 | Site_4 |
| SN10167 | *Anemone rivularis* Buchanan-Hamilton ex de Candolle | Ranunculaceae | 3657 | Site_4 |
| SN10168 | *Anemone trullifolia* J. D. Hooker & Thomson | Ranunculaceae | 3657 | Site_4 |
| SN10169 | *Campanula crenulata* Franchet | Campanulaceae | 3657 | Site_4 |
| SN10170 | *Cyananthus hookeri* C. B. Clarke in J. D. Hooker | Campanulaceae | 3657 | Site_4 |
| SN10171 | *Cynoglossum amabile* Stapf & J. R. Drummond | Boraginaceae | 3657 | Site_4 |
| SN10172 | *Gentiana yunnanensis* Franchet | Gentianaceae | 3657 | Site_4 |
| SN10173 | *Microula forrestii* (Diels) I. M. Johnston | Boraginaceae | 3657 | Site_4 |
| SN10174 | *Pedicularis cephalantha* Franchet ex Maximowicz | Orobanchaceae | 3657 | Site_4 |
| SN10175 | *Polygonum macrophyllum* D. Don | Polygonaceae | 3657 | Site_4 |
| SN10176 | *Polygonum nepalense* Meisner | Polygonaceae | 3657 | Site_4 |
| SN10177 | *Potentilla lineata* Treviranus | Rosaceae | 3657 | Site_4 |
| SN10178 | *Primula beesiana* Forrest | Primulaceae | 3657 | Site_4 |
| SN10179 | *Ranunculus yunnanensis* Franchet | Ranunculaceae | 3657 | Site_4 |
| SN10180 | *Salvia trijuga* Diels | Lamiaceae | 3657 | Site_4 |
| SN10181 | *Saxifraga strigosa* Wallich ex Seringe in de Candolle | Saxifragaceae | 3657 | Site_4 |
| SN10182 | *Stellera chamaejasme* Linnaeus | Thymelaeceae | 3657 | Site_4 |
| SN10183 | *Swertia macrosperma* (C. B. Clarke) C. B. Clarke in J. D. Hooker | Gentianaceae | 3657 | Site_4 |
| SN10184 | *Taraxacum eriopodum* (D. Don) Candolle | Asteraceae | 3657 | Site_4 |
| SN10185 | *Taraxacum sinicum* Kitagawa | Asteraceae | 3657 | Site_4 |
| SN10186 | *Adenophora coelestis* Diels | Campanulaceae | 3896 | Site_5 |
| SN10187 | *Aletris pauciflora* var. *khasiana* (J. D. Hooker) F. T. Wang & Tang | Liliaceae | 3896 | Site_5 |
| SN10188 | *Allium mairei* H. Léveillé | Liliaceae | 3896 | Site_5 |
| SN10189 | *Androsace spinulifera* (Franchet) R. Knuth in Engler | Primulaceae | 3896 | Site_5 |
| SN10190 | *Anemone trullifolia* J. D. Hooker & Thomson | Ranunculaceae | 3896 | Site_5 |
| SN10191 | *Arenaria leptophylla* | Caryophyllaceae | 3896 | Site_5 |
| SN10192 | *Campanula crenulata* Franchet | Campanulaceae | 3896 | Site_5 |
| SN10193 | *Codonopsis bulleyana* Forrest ex Diels | Campanulaceae | 3896 | Site_5 |
| SN10194 | *Comastoma cyananthiflorum* (Franchet) Holub, Folia Geobot. | Gentianaceae | 3896 | Site_5 |
| SN10195 | *Corydalis delavayi* Franchet | Papavaraceae | 3896 | Site_5 |
| SN10196 | *Cremanthodium nobile* (Franchet) Diels ex H. Léveillé | Asteraceae | 3896 | Site_5 |
| SN10197 | *Cyananthus macrocalyx* Franchet | Campanulaceae | 3896 | Site_5 |
| SN10198 | *Gentiana yunnanensis* Franchet | Gentianaceae | 3896 | Site_5 |
| SN10199 | *Halenia elliptica* D. Don | Gentianaceae | 3896 | Site_5 |
| SN10200 | *Hippolytia delavayi* (Franchet ex W. W. Smith) C. Shih | Asteraceae | 3896 | Site_5 |
| SN10201 | *Pedicularis cephalantha* Franchet ex Maximowicz | Orobanchaceae | 3896 | Site_5 |
| SN10202 | *Polygonum macrophyllum* D. Don | Polygonaceae | 3896 | Site_5 |
| SN10203 | *Potentilla peduncularis* D. Don | Rosaceae | 3896 | Site_5 |
| SN10204 | *Rhododendron fastigiatum* Franchet | Apiaceae | 3896 | Site_5 |
| SN10205 | *Saxifraga melanocentra* Franchet | Saxifragaceae | 3896 | Site_5 |
| SN10206 | *Stellera chamaejasme* Linnaeus | Thymelaeceae | 3896 | Site_5 |
| SN10207 | *Viola biflora var. rockiana* (W. Becker) Y. S. Chen | Violaceae | 3896 | Site_5 |

**Table S3:** Correlation coefficients of the phenotypic traits.

|  | **Pol_no** | **Ovu_no** | **P/O** | **Dis_area** | **Tb_dpth** | **Sti_ht** | **Sta_ht** | **Sep** | **Fl_no** | **Infl_ht** |
| --- | --- | --- | --- | --- | --- | --- | --- | --- | --- | --- |
| **Pol_no** | 1 |  |  |  |  |  |  |  |  |  |
| **Ovu_no** | -0.071 | 1 |  |  |  |  |  |  |  |  |
| **P/O** | **0.958**** | -0.12 | 1 |  |  |  |  |  |  |  |
| **DisA** | 0.047 | 0.275 | -0.117 | 1 |  |  |  |  |  |  |
| **Tb_dph** | -0.141 | 0.008 | -0.088 | 0.119 | 1 |  |  |  |  |  |
| **Sti_ht** | -0.191 | -0.03 | -0.17 | 0.227 | **0.824**** | 1 |  |  |  |  |
| **Sta_ht** | -0.207 | -0.05 | -0.2 | 0.304 | **0.736**** | **0.864**** | 1 |  |  |  |
| **Sep** | -0.102 | -0 | -0.094 | 0.044 | **0.59**** | **0.661**** | 0.271 | 1 |  |  |
| **Fl_no** | **0.692**** | -0.17 | **0.74**** | -0.16 | -0.03 | -0.09 | -0.132 | -0.035 | 1 |  |
| **Infl_ht** | 0.092 | 0.042 | 0.08 | 0.216 | **0.311** | 0.245 | 0.174 | 0.277 | 0.2 | 1 |

Pol_no = Pollen number per flower; Ovu_no = Ovule number per flower; P/O = Pollen ovule ratio per flower; Dis_area = Display area of the flower; Tb_dpth = Tube depth; Sti_ht = Stigma height; Sta_ht = Stamen height; Sep = Stigma-stamen separation; Fl_no = Flower number per individual; and Infl_ht = Inflorescence height. **Correlation at the ≤0.05 level are presented in bold.

**Table S4**: Maximum likelihood tests (95% of Confidence Interval) comparing phylogenetic generalized least square (PGLS) regression and Ordinary Least Square (OLS) regression for the number of pollen grains per flower (Pollen) on a series of phenotypic floral traits. Significant effects (*p* ≤ 0.05) are presented in bold.

| **Quantitative variables** | **PGLS (Maximum likelihood)** | | | | | **OLS** | | |
| --- | --- | --- | --- | --- | --- | --- | --- | --- |
|  | **AIC** | **BIC** | **logLik** | **t-value** | ***p*-value** | **AIC** | **t-value** | ***p*-value** |
| Pollen ~ Ovule | 136.03 | 143.32 | -65.01 | 5.33 | **<0.001** | 187.90 | 4.60 | **<0.001** |
| Pollen ~ P/O | 127.98 | 135.27 | -60.99 | 6.29 | **<0.001** | 166.36 | 7.16 | **<0.001** |
| Pollen ~ Display area | 153.06 | 160.35 | -73.53 | 2.86 | **<0.001** | 186.87 | 4.07 | **0.001** |
| Pollen ~ Tube depth | 156.69 | 163.99 | -75.35 | -2.08 | **<0.05** | 175.57 | -2.90 | **<0.05** |
| Pollen ~ Stamen height | 158.96 | 166.26 | -76.48 | -1.43 | 0.16 | 155.90 | -1.21 | 0.23 |
| Pollen ~ Stigma height | 157.84 | 165.13 | -75.92 | -1.78 | 0.078 | 158.40 | -1.21 | 0.23 |
| Pollen ~ Stigma stamen separation | 160.40 | 167.69 | -77.20 | 0.78 | 0.44 | 204.83 | 1.53 | 0.13 |
| Pollen ~ Flower number | 152.60 | 159.89 | -73.30 | -2.94 | **<0.05** | 202.27 | -0.40 | 0.69 |
| Pollen ~ Inflorescence height | 158.31 | 165.60 | -76.15 | -1.64 | 0.11 | 201.92 | 0.71 | 0.48 |
| **Qualitative variables** | **PGLS** | | | | | **ANOVA** | | |
|  | **AIC** | **BIC** | **logLik** | **t-value** | ***p*-value** | **Df** | **t-value** | ***p*-value** |
| Pollen ~ Flower shape | 156.67 | 163.97 | -75.34 | 2.09 | **<0.05** | 1 | 13.16 | **<0.001** |
| Pollen ~ Flower cluster | 161.02 | 168.31 | -77.51 | 0.03 | 0.98 | 1 | 3.21 | 0.07 |
| Pollen ~ Flower symmetry | 160.93 | 168.22 | -77.46 | 0.30 | 0.76 | 1 | 0.14 | 0.71 |
| Pollen ~ Pollen presentation | 157.28 | 164.58 | -75.64 | 1.93 | **0.05** | 1 | 13.90 | **<0.001** |

**Table S5:** Maximum likelihood tests (95% of Confidence Interval) comparing phylogenetic generalized least square (PGLS) and Ordinary Least Square (OLS) regression for ovule number per flower (Ovule) on a series of selected floral traits. Significant effects (*p* ≤ 0.05) are presented in bold.

| **Quantitative variables** | **PGLS (Maximum likelihood)** | | | | | **OLS** | | |
| --- | --- | --- | --- | --- | --- | --- | --- | --- |
|  | **AIC** | **BIC** | **logLik** | **t-value** | ***p*-value** | **AIC** | **t-value** | ***p*-value** |
| Ovule ~ P/Os | 120.18 | 127.47 | -57.09 | -4.16 | **<0.001** | 152.79 | -4.15 | **<0.001** |
| Ovule ~ Display area | 129.92 | 137.21 | -61.96 | 2.53 | **0.013** | 133.84 | 6.90 | **<0.001** |
| Ovule ~ Flower number | 127.15 | 134.44 | -60.57 | -3.06 | **<0.005** | 152.45 | -4.29 | **<0.001** |
| Ovule ~ Tube depth | 130.87 | 138.17 | -62.44 | -2.33 | **<0.05** | 133.68 | 0.57 | 0.57 |
| Ovule ~ Stamen height | 135.71 | 143.00 | -64.85 | -0.73 | 0.47 | 130.56 | 0.89 | 0.38 |
| Ovule ~ Stigma height | 135.37 | 142.66 | -64.69 | -0.93 | 0.36 | 168.48 | 0.55 | 0.59 |
| Ovule ~ Stigma stamen separation | 135.95 | 143.24 | -64.97 | 0.54 | 0.59 | 146.25 | -4.30 | **<0.001** |
| Ovule ~ Inflorescence height | 136.24 | 143.54 | -65.12 | -0.07 | 0.94 | 163.25 | -0.01 | 0.99 |
| **Qualitative variables** | **PGLS** | | | | | **ANOVA** | | |
|  | **AIC** | **BIC** | **logLik** | **t-value** | ***p*-value** | **Df** | **t-value** | ***p*-value** |
| Ovule ~ Flower shape | 131.07 | 138.36 | -62.53 | -2.28 | **<0.05** | 1 | 11.83 | **<0.001** |
| Ovule ~ Flower cluster | 136.25 | 143.54 | -65.13 | -0.02 | 0.99 | 1 | 0.00 | 0.94 |
| Ovule ~ Flower symmetry | 135.37 | 142.66 | -64.69 | -0.93 | 0.36 | 1 | 2.37 | 0.13 |
| Ovule ~ Pollen presentation | 136.09 | 143.39 | -65.05 | 0.39 | 0.70 | 1 | 0.00 | 0.97 |


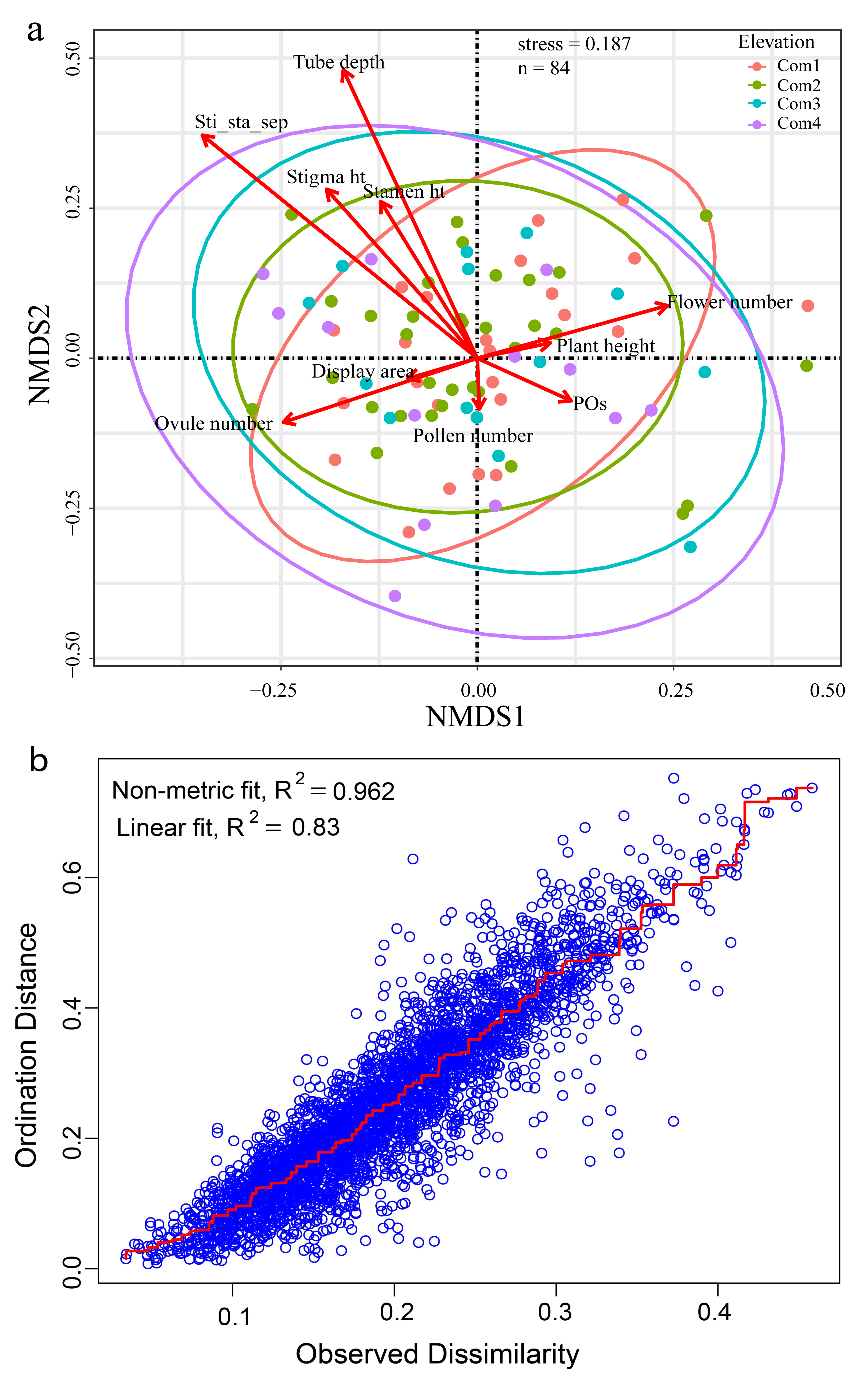


**Figure S1**: Non-metric Multidimensional Scaling (NMDS) Analysis. a. ordination of plant and flower traits among four different communities; stress value and sample size are presented in the graph; each community is represented by a different color. b. stress plot of the NMDS analysis showing the positive relationship between ordination distance and the observed dissimilarity.


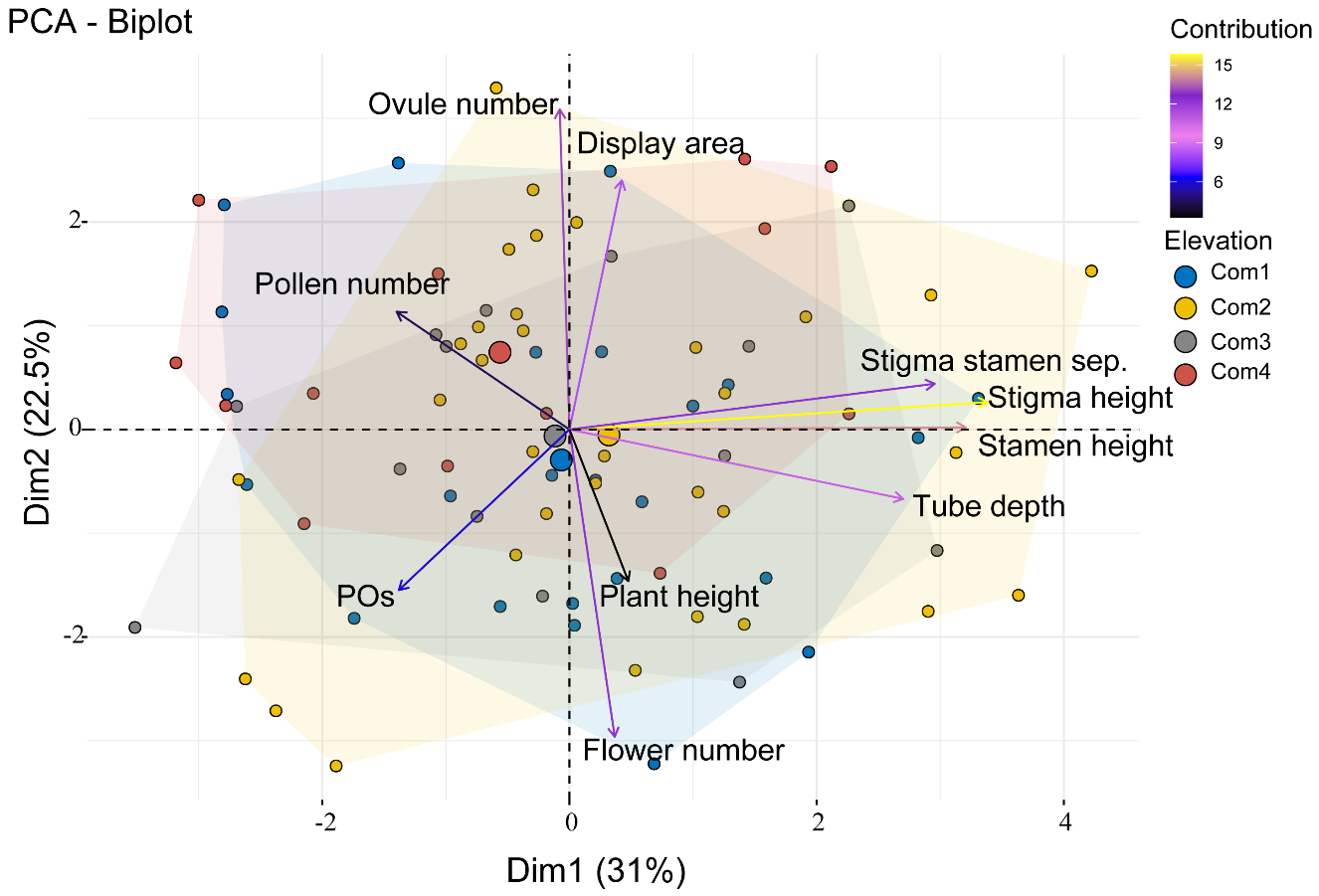


**Figure S2:** Principal component analysis (PCA) biplot representing phenotypic quantitative traits across the five different meadow communities. Each community (Com) is presented with a different color of the circles (see legend Elevation). The direction and length of the arrows associated with different color in the graph (see legend Contribution) represents the strength that each trait contributes to overall variance to visualize the strength that each trait contributes to explaining overall phenotypic variance across the communities, i.e., the higher a trait’s contribution the more this trait varies across communities; yellow represents the highest contribution where black indicates no significant contribution.


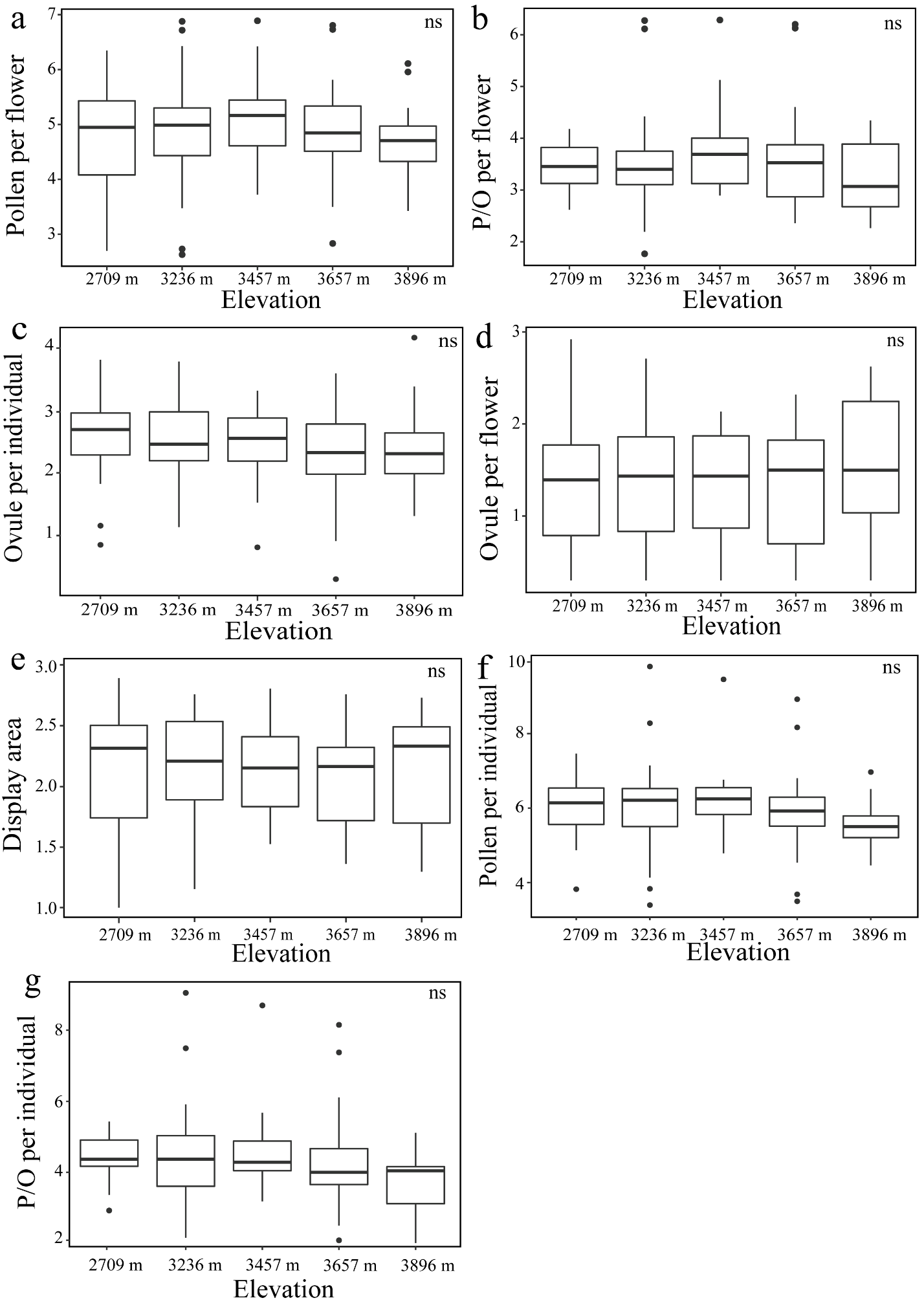


**Figure S3:** Non-phylogenetic analysis of all five-meadow communities located along an elevation gradient on Yulong Mountain, China. Plots representing the effect of elevation on a. pollen number, b. P/O per flower, c. ovule number per individual, d. ovule number per flower, e. flower display area, f. pollen number per individual, g. P/O per individual.
